# A novel biotinylated nanobody-based blocking ELISA for the rapid and sensitive clinical detection of porcine epidemic diarrhea virus

**DOI:** 10.1186/s12951-019-0531-x

**Published:** 2019-09-16

**Authors:** Zhiqian Ma, Tianyu Wang, Zhiwei Li, Xuyang Guo, Yangsheng Tian, Yang Li, Shuqi Xiao

**Affiliations:** College of Veterinary Medicine, Northwest A&F University, Yangling, 712100 Shaanxi China

**Keywords:** Biotinylated-nanobody, Blocking ELISA, PEDV

## Abstract

**Background:**

Porcine epidemic diarrhea virus (PEDV), which is characterized by severe watery diarrhea, vomiting, dehydration and a high mortality rate in piglets, leads to enormous economic losses to the pork industry and remains a large challenge worldwide. Thus, a rapid and reliable method is required for epidemiological investigations and to evaluate the effect of immunization. However, the current diagnostic methods for PEDV are time-consuming and very expensive and rarely meet the requirements for clinical application. Nanobodies have been used in the clinic to overcome these problems because of the advantages of their easy expression and high level of stability. In the present work, a novel biotinylated nanobody-based blocking ELISA (bELISA) was developed to detect anti-PEDV antibodies in clinical pig serum.

**Results:**

Using phage display technology and periplasmic extraction ELISA (PE-ELISA), anti-PEDV N protein nanobodies from three strains of PEDV were successfully isolated after three consecutive rounds of bio-panning from a high quality phage display VHH library. Then, purified Nb2-Avi-tag fusion protein was biotinylated in vitro. A novel bELISA was subsequently developed for the first time with biotinylated Nb2. The cutoff value for bELISA was 29.27%. One hundred and fifty clinical serum samples were tested by both newly developed bELISA and commercial kits. The sensitivity and specificity of bELISA were 100% and 93.18%, respectively, and the coincidence rate between the two methods was 94%.

**Conclusions:**

In brief, bELISA is a rapid, low-cost, reliable and useful nanobody-based tool for the serological evaluation of current PEDV vaccines efficacy and indirect diagnosis of PEDV infection.

## Background

Porcine epidemic diarrhea virus (PEDV) is the causative agent of porcine epidemic diarrhea (PED), a highly contagious enteric disease characterized by severe watery diarrhea, vomiting and dehydration leading to enormous economic losses in the swine industry worldwide [1, 2]. After PEDV was first reported in Belgium in 1971 and the United Kingdom in 1978, it then emerged in other European countries over the following several years [3]. PEDV can infect pigs of all ages and causes high mortality in newborn piglets (mortality rate is nearly 100%); however, PEDV mainly causes slow growth in adult pigs. In December 2010, a newly emerged and high virulent strain of PEDV rapidly spread in China, where it killed over 1,000,000 piglets with a mortality rate in suckling piglets approaching 100% [4, 5]. PEDV was identified as a member of the genus *Alphacoronavirus* within the family *Coronaviridae* in the order *Nidovirales* [6]. It is an enveloped, single-stranded, positive-sense RNA virus with a genome approximately 28 kb in length that comprises at least seven open reading frames (ORFs) encoding nonstructural ORF1a, ORF1b and ORF3 proteins and the structural spike (S), envelope (E), membrane (M) and nucleocapsid (N) [7]. One of the four structural proteins, the N protein, which is associated with viral replication, transcription and assembly, is a basic internal phosphoprotein important for inducing cell-mediated immunity in the host [8, 9]. Pigs produce high levels of antibodies against the N protein in the early stages of PEDV infection [6, 8]. Anti-N protein IgG antibodies were first detected on day 7 post infection, so the PEDV N protein is the best candidate antigen for early diagnosis because this gene is highly conserved [10].

In recent decades, a variety of methods to detect PEDV have been developed and reported in numerous studies. Since the clinical signs and histological changes in PED and other diarrheal diseases, such as transmissible gastroenteritis (TGE), are similar, they cannot be diagnosed without molecular methods and immunoassays [11, 12]. Conventional PEDV diagnostic methods are based on laboratory tests and include virus isolation, conventional reverse transcription-polymerase chain reaction (RT-PCR) [13, 14], real-time RT-PCR [15–17], indirect fluorescent antibody (IFA) assay [18] and enzyme-linked immunosorbent assay (ELISA) [19]. However, these conventional methods are time-consuming, and expensive, exhibit low specificity and sensitivity, and require well-trained technicians and special instruments. Moreover, issues such as false-positive results may arise from cross-contamination between samples or transportation delays [20]. Currently, different types of ELISAs, including indirect [19, 21], competitive and blocking ELISA, have been widely applied to detect PEDV in large-scale blood or feces samples, but these assays are based on the use of PEDV-specific monoclonal or polyclonal antibodies that require more support cost and exhibit low expression yields and high levels of instability [22].

Antibody-mediated immune detection is a popular approach due to its convenience. Nanobodies, also termed the variable domain of heavy-chain only antibody (VHH), were surprising discovered in the sera of camelids, such as llamas, dromedaries, camels, alpaca and vicuna [23, 24]. Nanobodies are also the smallest antibodies with complete antigen-binding sites [25]. The single-domain nature of nanobodies due to their lack of light chains confers many special properties not observed in conventional antibodies: including high affinity, thermal stability and high yield in microbial production systems [26, 27]. Moreover, because the molecular weights of nanobodies are only approximately 15 kDa and they are associated with concave epitopes, nanobodies might be better adapted to access hidden targets and cryptic sites than antibodies [22]. Based on these unique features, nanobodies hold great potential as candidates for diverse biomedical applications, such as disease diagnosis and therapeutics [28].

PEDV-specific nanobodies have not yet been reported; however, we motivated to use phage display technology to obtain the special nanobodies against the N protein of PEDV, and the selected nanobody was applied to develop a blocking ELISA for the rapid, low-cost and sensitive detection of PEDV in serum samples. Overall, this detection system will be extremely useful for future PEDV diagnosis.

## Materials and methods

### Serum samples

Information on serum samples used in this study is available in the materials and methods section of an Additional file 1.

### Camel immunization and library construction

A 4-year-old male Bactrian camel was immunized with recombinant PEDV N protein as described in previous studies with modifications; peripheral blood lymphocytes (PBLs) were then isolated from fresh blood as described in detail in Additional file 1 [28, 29]. Total RNA was isolated from PBLs using RNAiso Plus (Takara, Japan) according to the manufacturer’s instructions, and the RNA was then reverse transcribed to cDNA using the SuperScript III First-Strand Synthesis System (Thermo Fisher, USA). The variable regions of the VHH were amplified by nested PCR using Prime STAR HS DNA polymerase (Takara, Japan) with previously described primers [30, 31]. The first PCR products contained a 700 bp fragment amplified with CALL001 and CALL002 primers (Sangon Biotech, China) (Additional file 1: Table S1), and an approximately 400 bp VHH fragment was PCR-amplified with the VHH-FOR and VHH-REV primers containing PstI and NotI sites (Additional file 1: Table S1) and the first purified product used as a template. The pCANTAB 5E phagemid vector (GE Healthcare Life Science, USA) and the purified VHH products were digested using the *Pst*I and *Not*I restriction enzymes (New England Biolabs, NEB) and then ligated at 16 °C with T4 DNA ligase (New England Biolabs, NEB). The ligated material was electroporated into electrocompetent *E. coli* TG1 cells, and then the transformed cells were cultured on Luria–Bertani (LB) agar plates containing 2% (v/v) glucose and 100 μg/mL ampicillin at 37 °C overnight. Colonies were scraped and stored in LB containing 15% glycerol at − 80 °C. The size and diversity of the library were evaluated by calculating the number of colonies and the insertion rate by PCR amplification after gradient dilution, and the positive clones were sequenced.

### Phage preparation

Recombinant *E. coli* TG1 containing the pCANTAB 5E-VHH plasmid was inoculated into 2× YT medium containing 2% (v/v) glucose and 100 μg/ml ampicillin with vigorous shaking at 200 r/min and 37 °C until the optical density at 600 nm (OD_600nm_) of the bacterial culture reached approximately 0.6. Then, the M13K07 helper phage was added to TG1 cells, which were incubated for 1 h at 37 °C without shaking. Cells were harvested by centrifugation at 2800×*g* for 10 min, and the cells were resuspended and grown overnight in 2 × YT medium containing 100 μg/mL ampicillin and 50 μg/mL kanamycin. Cell culture was collected by centrifugation at 3800×*g* for 30 min at 4 °C, and the supernatant was mixed with PEG/NaCl. Phages were precipitated by centrifugation at 12,000×*g* for 30 min after 2 h of incubation on ice. Finally, the phage particles were resuspended in 1 mL of phosphate buffered saline (PBS) and then quantified by phage titration. The remaining phages were stored at − 80 °C for subsequent experiments.

### Phage titration

One hundred microliters of phage particles dissolved in PBS was serially diluted tenfold in 2× YT medium, and 100 µl of each suspension containing 10^−8^, 10^−9^ and 10^−10^ cells was added to 100 µl of TG1 cells and incubated for a half-hour at 37 °C without shaking. Then, the transformed cells were cultured on LB agar plates containing 2% (v/v) glucose and 100 μg/ml ampicillin at 37 °C overnight. The next day, plates containing approximately 50 to 150 colonies were selected, and the phage was quantified with the following formula: the titer value = number of colonies × dilution × 10.

### Panning for a special VHH against the PEDV N protein

To select special nanobodies against the PEDV N protein, three consecutive rounds of bio-panning were performed with the target protein. A 96-well plate was coated with PEDV N protein (100 µg/ml) diluted in PBS (100 µl/well) overnight at 4 °C, and PBS under the same conditions was used as a control. On the next day, the panning plate was blocked with 2.5% (w/v) nonfat dried milk (200 µl/well) in PBS (MPBS) at 25 °C for 1 h. Blocked wells were washed four times with PBST (PBS with 0.05% Tween-20 (v/v)). Then, the resuspended phage particles, which were diluted in MPBS, were prepared at a concentration of 4.4 × 10^11^ pfu/ml and incubated in panning plates (100 µl/well) for 1 h at 25 °C. The wells were then washed with PBST ten times and PBS five times. The special phage particles were eluted with 100 mM triethylamine (100 µl/well) (Sigma, USA) for 10 min at 25 °C and the supernatant was transferred to a 1.5 ml tube, following which 1 M Tris–HCl (pH 7.4) was added to neutralize the solution. Then, a 20 ml aliquot of fresh exponentially growing *E. coli* TG1 culture was infected with a 1.5 ml aliquot of eluted phage particles for a half-hour at 37 °C without shaking, followed by incubation in 80 ml 2× YT medium containing 2% (v/v) glucose and 100 μg/ml ampicillin with vigorous shaking at 200 r/min and 37 °C until the OD_600nm_ of the bacteria culture reached approximately 0.6. The M13K07 helper phage was added to the TG1 cells to rescue and enrich the special phage particles by three consecutive rounds of bio-panning with the procedures described above. Enrichment in each round of panning was assessed by phage titration, ELISA and colony PCR.

### Detecting antigen-specific antibodies by phage ELISA

A total of 98 different colonies were randomly selected from the third round of panning and cultured in 96-well plates containing 100 µl of LB agar, 2% (v/v) glucose and 100 μg/mL ampicillin for 8 h at 37 °C. Then, 20 µl of each cloned culture was transferred to 1 ml of Terrific Broth (TB) in 24-well plates and incubated with vigorous shaking at a speed of 200 r/min at 37 °C until the OD_600nm_ of the bacterial cultures reached approximately 0.6. Then, 100 µl of 10 mM isopropyl-β-d-thiogalactopyranoside (IPTG) was added to the TB to induce VHH antibody expression, and the culture was incubated overnight at 37 °C. The antibodies were extracted using an osmotic shock protocol and identified by periplasmic extract ELISA (PE-ELISA).

A 96-well plate was coated with PEDV N protein (100 µg/ml) diluted in PBS (100 µl/well) overnight at 4 °C, and PBS under the same conditions was used as a control. On the next day, the wells were blocked with 2.5% (w/v) MPBS at 37 °C for 1 h. Then, 100 µl of extracted antibodies (diluted 1:1 in MPBS) was added to each well and incubated for 1 h. Afterward, 100 µl of rabbit anti-E-tag polyclonal antibody (diluted 1:2000 in MPBS) was added to each well and incubated at 37 °C for 1 h. The plate was washed four times with PBST, and then 100 µl of goat anti-rabbit monoclonal antibody conjugated to HRP (diluted 1:5000 in MPBS) (TransGen Biotech, China) was then added and incubated for 1 h at 37 °C. After another washing step, 100 µl of a fresh TMB (400 µl of 0.6% TMB and 100 µl of 1% H_2_O_2_ in 25 ml citrate buffer, pH 5.5) solution was added to the plate, which was incubated at 37 °C for 30 min. The reaction was stopped by the addition of 50 µl of 3 M H_2_SO_4_. The absorbance at 450 nm was read on a microtiter plate reader (Bio-Rad, USA). If the absorbance in the antigen-coated well was at least threefold greater than that of the well containing PBS, the colony was regarded as positive and subjected to sequence analysis (Sangon Biotech, China).

### Detecting the specificity, binding activity and affinity of VHH antibodies

The specificity and binding activity of PEDV N protein nanobodies were determined by indirect ELISA. Specificity was determined according to the method described above, with the TGEV N protein applied as a control. The binding activity was assessed with the same method; in this assay, positive nanobodies and unrelated nanobodies were diluted by multiple factors.

In order to verify the affinity of PEDV N antigen binding to the Nbs, we conducted surface plasmon resonance (SPR) analysis using a Biacore 3000 instrument (GE Healthcare Life Science, USA).The experimental procedure is as follows: the purified PEDV N protein (5 µg/ml) was immobilized onto the flow cell of a CM5 sensor chip (GE Healthcare Life Science, USA) using a standard amine-immobilization kit as specified by the manufacturer (Biacore). The SPR assay was performed using HBS-EP running buffer (10 mM HEPES, pH 7.4, 150 mM NaCl, 3 mM EDTA, and 0.005% (v/v) surfactant P20) on the Biacore 3000 instrument at room temperature. The Nb1, Nb2 and Nb3 of same concentration (3 µg/ml) were flowed over the chip surface respectively. The chips were regenerated with glycine–HCl buffer (pH 2.0). The sensorgrams were analyzed using BIA evaluation Software version 4.1. A nanobody sample exhibiting good specificity, binding activity and affinity was selected for the subsequent experiment.

### Expression and purification of soluble nanobody

The gene encoding the PEDV N protein specific nanobody was amplified with the pET21b-VHH-F and pET21b-VHH-R primers (Sangon Biotech, China) containing *Bam*HI and *Hin*dIII sites and an Avi-tag (Additional file 1: Table S1) and then cloned into the pET21b expression vector. The recombinant plasmids were transformed into *E. coli* BL21(DE3) to express nanobodies. The cells were grown in LB at 200 r/min and 37 °C until the OD_600nm_ of the bacterial culture reached approximately 0.6, following which expression was induced using 1 mM IPTG followed by incubation at 200 r/min and 37 °C for 5 h. The cells were collected through centrifugation and ultrasonically treated. The soluble protein was suspended in PBS and purified with Ni-NTA according to the manufacturer’s instructions (GE Healthcare Life Science, USA). The purified soluble protein at a final concentration of 1 mg/ml was biotinylated using biotin ligase (GeneCopoeia, China) according to the manufacturer’s instructions.

### Cross-reactivity assay

The specificity of the bio-nanobody was tested by indirect ELISA, and six different viral proteins, (the PEDV N, PEDV S, TGEV N, PRRSV N, PCV Cap and PRV gE proteins) were chosen to test potential cross-reactivity. The same amount of the six proteins were used to coated a 96-well plate and incubated overnight at 4 °C. After blocking, the biotinylated-nanobody was diluted to different concentrations and incubated with antigen at 37 °C for 1 h. Then, HRP-conjugated streptavidin (diluted 1:5000 in MPBS) was added and incubated at 37 °C for 30 min. After another washing step, 100 µl of a fresh TMB solution prepared as described above was added to the plate and incubated at 37 °C for 15 min. The reaction was stopped by the addition of 50 µl of 3 M H_2_SO_4_, and then the absorbance at 450 nm was read on a microtiter plate reader.

### Procedures of the for streptavidin–biotin amplified blocking ELISA

Biotinylated nanobody blocking ELISA (bELISA) was performed with a streptavidin–biotin amplified system. To achieve optimal blocking ELISA performance, various experimental conditions were optimized. First, a checkboard titration was implemented in indirect ELISA to determine the optimal concentration of the antigen (50 ng/well, 100 ng/well, 200 ng/well, and 400 ng/well) and dilution factor of the corresponding biotinylated nanobody (1:500, 1:1000, 1:2000, 1:4000, 1:6000 and 1:8000). If the absorbance of the solution in the antigen-coated well was close to 1.0, the concentration of the corresponding coated antigen and the dilution factor of the biotinylated nanobody were considered optimal.

To carry out bELISA, a 96-well plate was coated with the optimal concentration of PEDV N protein, incubated overnight at 4 °C and then blocked with 1% BSA at 37 °C for 1 h. The positive and negative sera were serially diluted (1:5, 1:10, 1:20, 1:40) and added to the wells at 37 °C for different lengths of time (30 min, 60 min, 90 min, 120 min). Then, the plate was washed four times with PBS’T. Afterward, the biotinylated nanobody was added to the wells and incubated for different lengths of time (30 min, 60 min, 90 min and 120 min). The plate was washed three times with PBST and then the optimal concentration of HRP-conjugated streptavidin (Thermo Fisher, USA) was added for incubation at 37 °C for the optimal reaction time (30 min, 60 min, 90 min and 120 min). After another washing step, 100 µl of a fresh TMB solution prepared as described above was added to the plate, which was incubated at 37 °C for 15 min. The reaction was stopped by the addition of 50 µl of 3 M H_2_SO_4_, and then the absorbance at 450 nm was read on a microtiter plate reader. If the percentage inhibition (PI) {PI (%) = 100 × [1 − (test serum OD_450nm_/negative reference serum OD_450nm_)]} was highest, and the absorbance of the negative serum was closest to 1.0, the experimental conditions were then considered optimal.

### Blocking ELISA cutoff value

Ninety standard PEDV-negative serum samples, that were confirmed PEDV-negative by both RT-PCR and a commercial ELISA kit were used to define the positive–negative cutoff value for blocking ELISA. The procedure was based on the method mentioned above. The two thresholds cutoff value were defined as the mean PI (%) value ($\overline{x}$) of the negative samples (N) + 2 or 3× standard deviations (SDs). Serum samples showing PI (%) values greater than or equal to $\overline{x}$ + 3SD were considered PEDV-seropositive. Serum samples showing PI (%) values between $\overline{x}$ + 2SD and $\overline{x}$ + 3SD were considered suspicious. Serum samples showing PI (%) values less than or equal to $\overline{x}$ + 2SD were considered PEDV-seronegative.

### Specificity, sensitivity and repeatability of bELISA

The specificity of biotinylated nanobody bELISA were evaluated. First, serum positive for other common pig pathogens, including TGEV, PPV, PRRSV, PRV, JEV, PCV2, and CSFV, was used for blocking ELISA analysis performed according to the above steps. These positive serums samples were confirmed PEDV-negative by using both RT-PCR and commercial ELISA kits. Then, five different viral proteins, the PEDV N, PEDV S, TGEV N, PCV Cap and PRV gE proteins, 100 ng/well were used to coat a 96-well plate overnight at 4 °C, and bELISA was performed according to the above steps.

To further evaluate the sensitivity of bELISA, standard positive and negative sera were diluted to different concentrations (1:5, 1:10, 1:20, 1:40 and 1:60) then subjected to bELISA.

The repeatability of bELISA was evaluated with four PEDV-seropositive samples and two PEDV-seronegative by determining the intra-assay variability. To determine intra-assay variability, each sample was tested 4 times by different operators on different occasions. The results are presented as the standard deviation (SD) of the percentage inhibition of each group of samples.

### Detection of field serum samples

Biotinylated nanobody bELISA was applied to evaluate field serum samples. A total of one hundred and fifty field serum samples with unknown PEDV exposure status were collected from 5 farms in the Shannxi and Shandong provinces from 2016 to 2018. The samples were analysis by bELISA as described above. To validate the results of biotinylated nanobody bELISA, the PEDV exposure status of the samples was also confirmed by commercial ELISA kits according to manufacturer’s instruction.

### Statistical analysis

Statistical analysis and drawing were performed using GraphPad Prism software 5.0 (GraphPad Software, Inc. LA Jolla, CA, USA). The Kappa values were calculated to estimate the coincidence between bELISA and the commercial ELISA kit using SPSS software (Version 20, http://www.spss.com.cn).

## Results

### Generation of the immunized library

**Fig. 1:**
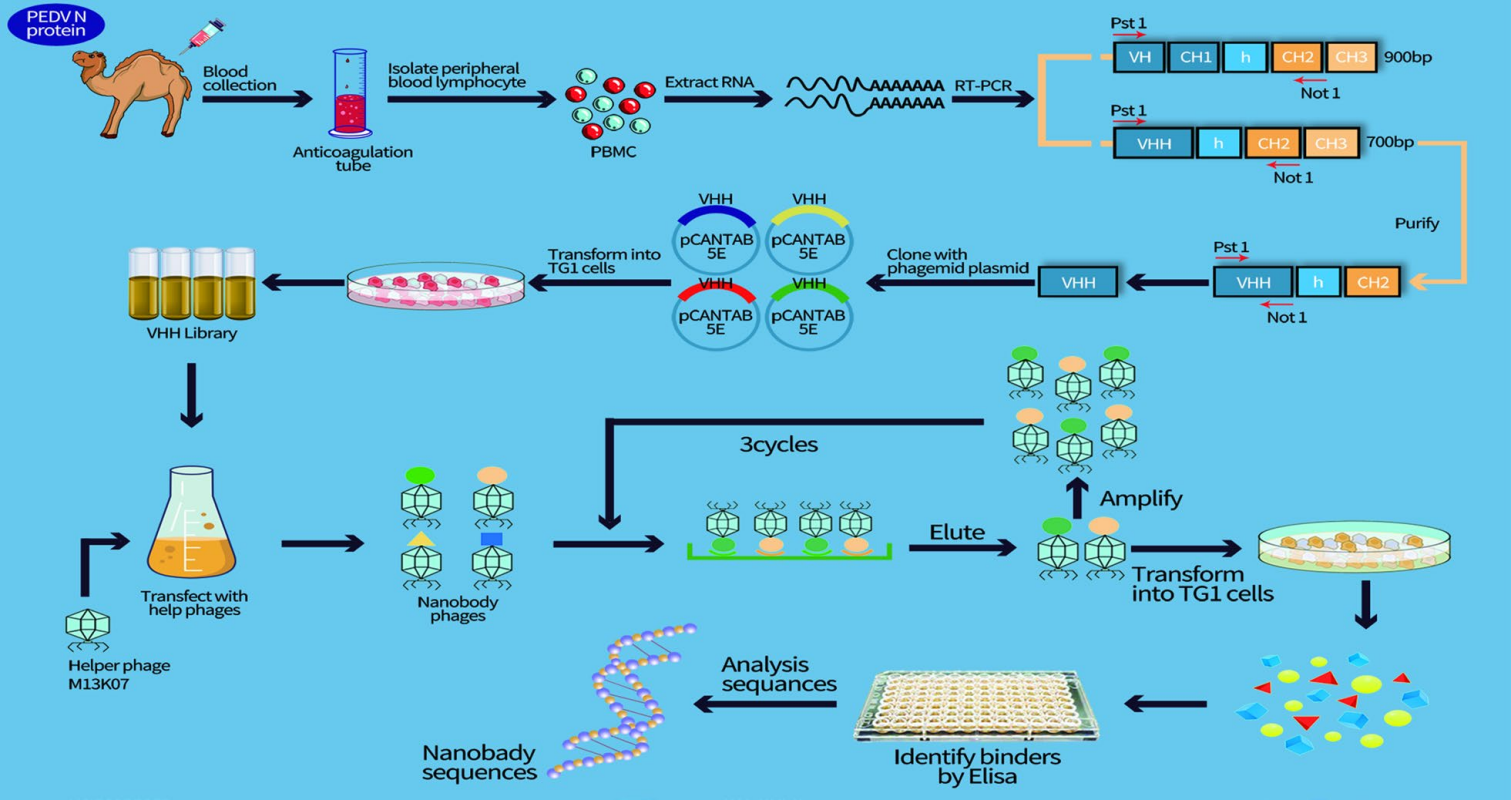
Flow diagram for the acquisition of specific nanobodies from an immunized phage display library

The strategy used to construct a VHH library is described in Fig. 1. To generate VHH-specific PEDV N protein, a 4-year-old healthy male Bactrian camel was immunized with highly pure N-His-tagged recombinant protein five times with 2-week intervals. Polyclonal antisera were extracted 4 days after the fifth immunization and used to evaluate the anti-N protein response by indirect ELISA. The viral titer of the antiserum collected following the fifth immunization reached 1:128,000, indicating that the antiserum produced a raised good immunogenic response (Additional file 1: Fig. S1). Total RNA was extracted from peripheral blood lymphocytes (PBLs), and then reverse transcribed to cDNA to construct the VHH library. cDNA was used as a template to amplify specific VHH fragments using nesting PCR. A 700 bp fragment in the first PCR product (Additional file 1: Fig. S2A) and a 400 bp fragment in the VHH PCR product were generated using the first purified product as a template (Additional file 1: Fig. S2B). The purified VHH product and the linearized pCANTAB 5E phagemid vector were ligated and electroporated into electrocompetent *E. coli* TG1 cells, and the transformed cells were then cultured on Luria–Bertani (LB) agar plates to generate the VHH library. The size of the library was approximately 6.3 × 10^8^ pfu/ml, which was evaluated by calculating the number of colonies after gradient dilutions. Ninety-six single colonies were randomly selected for bacterial fluid PCR, and eleven single positive colonies were then randomly selected for sequencing. The recombination rate of the library was approximately 88.5%, and the amino acid sequence homology was approximately 45.5%, indicating the rich diversity of the library (Additional file 1: Fig. S2C). The titer of the recombinant phage library reached 4.4 × 10^13^ pfu/ml after rescue of the constructed library with the M13KO7 helper phage.

### Bio-panning and phage ELISA analysis

**Fig. 2:**
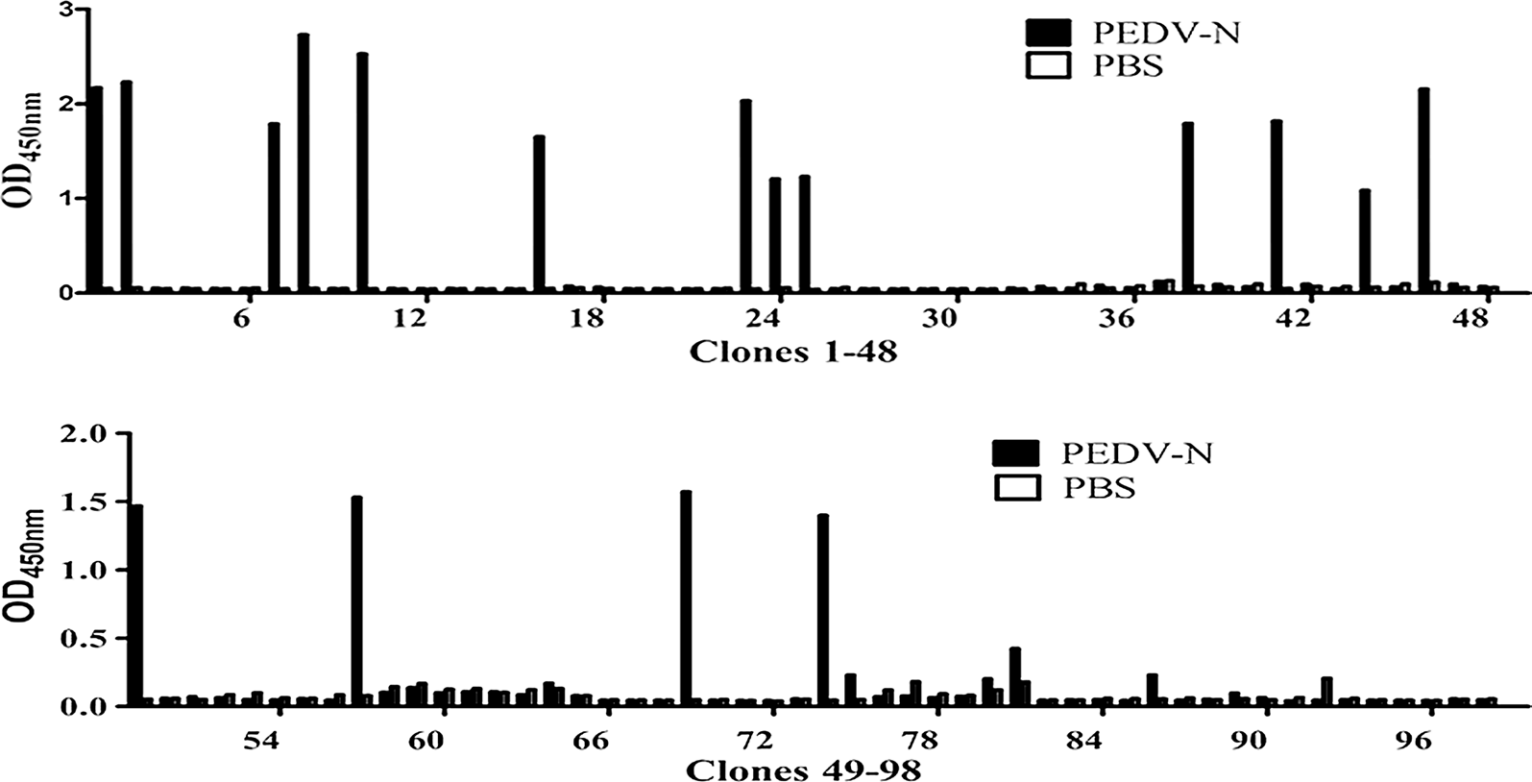
Identification of PEDV N protein specific nanobodies. A periplasmic extract ELISA detected 98 clones, of which 19 clones were identified as positive. The absorbance of the reaction mixture in an antigen-coated well was at least threefold greater than that of the reaction mixture in a PBS-coated well and considered positive

**Fig. 3:**
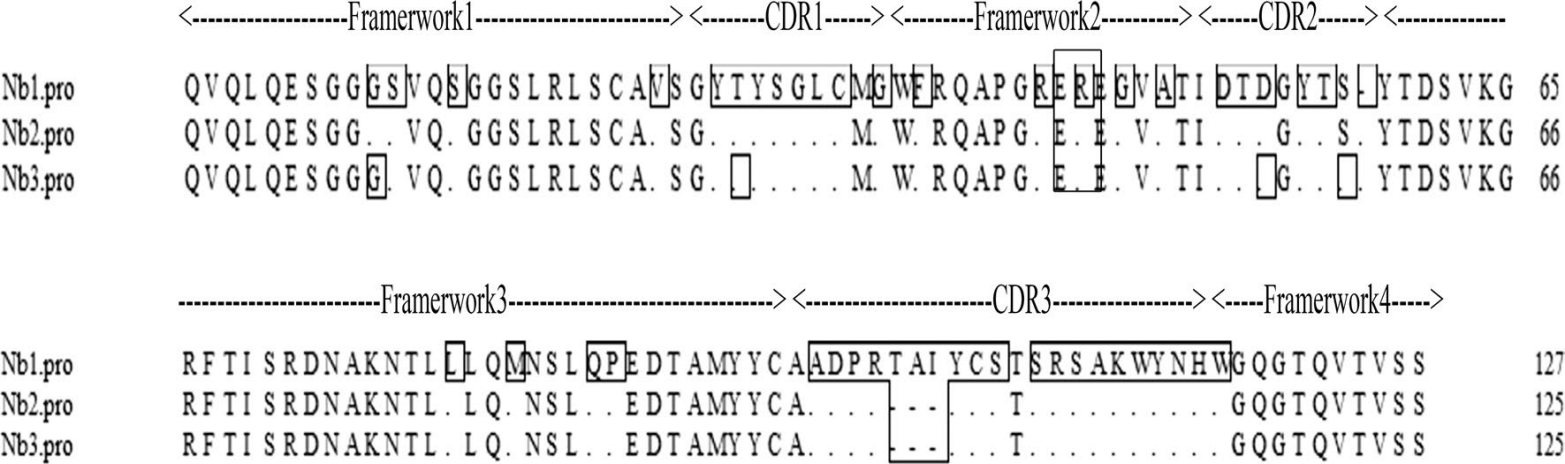
Alignment of the amino acid sequences of the selected Nb1, Nb2 and Nb3 nanobodies. The sequences are grouped according to the similitude of their CDRs. Dots (as‘.’) indicate residues that differ from those in Nb1. Boxes are used to indicate residues that differ from those in Nb2

To obtain highly specific nanobodies against the PEDV N protein, three consecutive rounds of bio-panning were carried out using the PEDV N protein or PBS as the antigen. The titer of the recombinant phage in the final eluent of each round of panning was tested to evaluate the enrichment of the specific phage. Compared with that observed for the negative control, the enrichment factors were 0.047-, 69-, and 1650-fold higher after the first, second, and third rounds of panning respectively, indicating the significantly enrichment of specific nanobodies during bio-panning (Table 1). Moreover, to obtain soluble nanobodies with high diversity and affinity, 98 clones randomly selected from each round were screened for PEDV N protein by periplasmic extraction followed by phage ELISA. The diversity of the ELISA-positive colonies was determined by sequencing. Plasmids were extracted from 19 clones that exhibited a positive reaction compared with that of the negative control (Fig. 2) and analyzed by sequencing. According to the amino acid sequence of the three highly variable complementary determination regions (CDR1, CDR2, and CDR3) of the clones, the nanobodies were divided into three groups: Nb1, Nb2, and Nb3 (Fig. 3). The complementary determination regions contained the typical hydrophilic amino acid substitutions in the framework of relatively conservative FR2, including a G/E substitution at position 44 of the three nanobodies and V/F, L/R and W/G substitution at positions 37, 45, and 47 of Nb1 respectively. In addition, the CDR1 and CDR3 regions of Nb1 also contained cysteine residues, which can form disulfide bonds, contribute to the formation of a ring structure and play an important role in stabilizing the structure of the antigen-binding region of the nanobody. This explains the absence of an interaction with the VL domain as well as the high solubility of VHH as a single-domain fragment [32, 33].

**Table 1:** The enrichment nanobodies in the VHH library were detected through three consecutive rounds of biopanning, and PEDV N specific nanobodies were enriched in the VHH library by approximately 1650 fold

Round of panning	Input phage (pfu/well)	P output (pfu/well)	N output (pfu/well)	Recovery (P/input)	P/N
1st round	4.4 × 10^11^	3.5 × 10^5^	7.3 × 10^4^	7.9 × 10^−7^	0.047
2nd round	2.0 × 10^11^	9.7 × 10^7^	1.4 × 10^6^	4.8 × 10^−5^	69
3rd round	5.2 × 10^11^	3.8 × 10^9^	2.3 × 10^6^	7.3 × 10^−3^	1.65 × 10^3^

To further evaluate the specificity, binding activity and affinity of the three nanobodies, PEDV N and TGEV N proteins were subjected to PE-ELISA, which revealed that Nb2 and Nb3 can specifically bind to the PEDV N protein without cross-reaction with the TGEV N protein (Additional file 1: Fig. S3A). The titers of three nanobodies and the unrelated nanobody Nb12 were also determined to further assess their binding activity, which indicated that Nb2 has a higher binding activity than Nb1 and Nb3 (Additional file 1: Fig. S3B).

The sensorgrams indicated that PEDV N protein interacted with Nb1, Nb2 and Nb3 respectively (Additional file 1: Fig. S4). Furthermore, we used the 1:1 Langmuir model and sensorgrams data for kinetic analysis of PEDV N protein and Nbs. The *Ka* values (1.97 × 10^5^ M^−1^ s^−1^, 9.21 × 10^5^ M^−1^ s^−1^ and 3.05 × 10^5^ M^−1^ s^−1^) indicated a fast association constant between Nb1, Nb2 and Nb3 respectively and PEDV N protein (Additional file 1: Table S2). The *Ka/Kd* values, the equilibrium dissociation constant between Nb1, Nb2 and Nb3 and PEDV N protein, measured for Nb1, Nb2 and Nb3 reached up to 0.96 × 10^8^ M, 5.32 × 10^8^ M and 1.89 × 10^8^ M respectively (Additional file 1: Table S2). Overall, our data suggested that Nb2 exhibits a highest binding affinity to the PEDV N protein. Therefore, Nb2 was selected and applied in further studies.

### Expression and purification of nanobodies

**Fig. 4:**
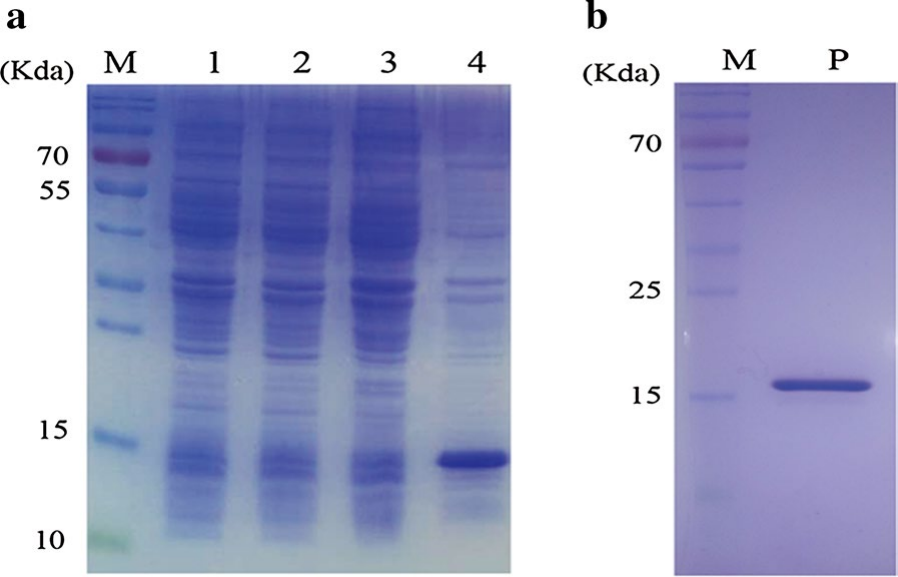
Expression and purification of recombination Nb2 protein. **a** SDS-PAGE and Coomassie brilliant blue staining analysis of the expression of recombination Nb2 protein nearly 15KDa in size. Lane M: Prestained protein marker. Lane1: whole protein lysate of bacteria transformed with uninduced empty vector. Lane2: whole protein lysate of induced bacteria transformed with empty vector. Lane3: whole protein lysate of uninduced bacteria transformed with Nb2. Lane4: whole protein lysate of induced bacteria transformed with Nb2. **b** Analysis of purified recombination Nb2 protein by SDS-PAGE and Coomassie brilliant blue staining. Lane M: prestained protein marker. P: purified Nb2 protein

The properties of nanobodies include their small size (approximately 15 kDa), high affinity, high level of stability and single-stranded composition, so nanobodies are easy to produce in microorganisms at a higher level than traditional antibodies. To produce the Nb2 protein, the Nb2-Avitag gene was cloned into the pET21b expression vector, and the recombinant ‘pET21b-Nb2-Avitag’ vector was then transformed into BL21 (DE3) cells. The recombinant Nb2-Avitag protein, which was approximately16 kDa, was expressed in inclusion bodies that were detected by 12% SDS-PAGE (Fig. 4a). Highly pure Nb2 protein was obtained by purification using a Ni–NTA affinity column, and the purity of Nb2 was detected by 12% SDS-PAGE (Fig. 4b). The Nb2-Avitag protein was renatured in PBS by continuous dialysis, and the biotinylated form of Nb2 was produced using biotin ligase in vitro, and is named BiNb2.

### Cross-reactivity assay

**Fig. 5:**
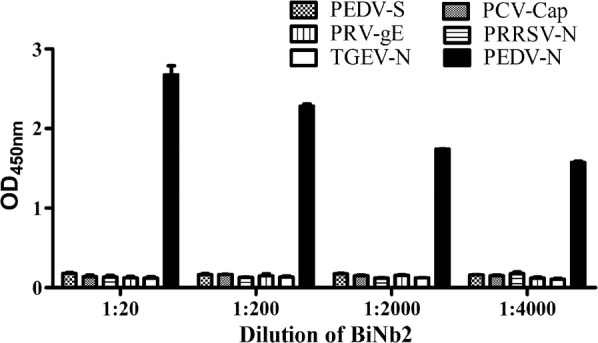
Specificity of purified biNb2. The specificity of purified biNb2 was tested by indirect ELISA in 96-well plates coated with different antigens as against the PEDV S, TGEV N, PRRSV N, PCV-Cap and PRV-gE proteins (100 ng/well). Serially diluted biNb2 in MPBS was added to the wells. Then, the optimal concentration of HRP-conjugated streptavidin was added. Each result is represented as an average ± standard deviation of three replicates

To ensure that BiNb2 is the specific to PEDV N protein, the reaction of BiNb2 with PEDV N, PEDV S, TGEV N, PRRSV N, PCV Cap and PRV gE proteins were examined by indirect ELISA. BiNb2 diluted 1:4000 bound to the PEDV N protein with high specificity. Moreover, BiNb2 even at high concentrations (1:20) exhibited no cross-reactivity with the other viral proteins (Fig. 5).

### Optimization of streptavidin–biotin amplified bELISA

The checkboard titration method was first carried out by indirect ELISA to determine the optimal concentration of the PEDV N protein (100 ng/well) and dilution factor for the corresponding antibody against BiNb2 (1:8000). Furthermore, various experimental conditions were optimized according to the bELISA procedure.

A 96-well plate was coated with 100 ng/well PEDV N protein and incubated overnight at 4 °C (Additional file 1: Fig. S5A). On the next day, the wells were blocked with 1% BAS at 37 °C for 1 h. Positive and the negative sera were diluted 1:5 and added to the wells, followed by incubation at 37 °C for 2 h (Additional file 1: Fig.S5B and Fig.S5C); then, the plate was washed three times with PBST. Afterward, the biotinylated nanobody was diluted at 1:8000, added to the wells and incubated at 37 °C for 30 min (Additional file 1: Fig. S5A, D). The plate was washed three times with PBST, and HRP-conjugated streptavidin was diluted 1:2000 and added to the to the plat before incubation at 37 °C for 1 h (Additional file 1: Fig.S5E). After another wash step, 100 µl of fresh TMB solution prepared as described above was added to the plate, which was incubated at 37 °C for 15 min. The reaction was stopped by the addition of 50 µl of 3 M H_2_SO_4_, and then the absorbance at 450 nm was read on a microtiter plate reader and used to calculate the blocking rate.

To determine the cutoff value of the bELISA, one hundred standard PEDV-negative serum samples were tested by bELISA. The average PI (%) ($\overline{x}$) of the negative serum samples was 2.29%, and the standard deviation (SD) of the blocking rates determined from these samples was 8.99%. Thus, the cutoff values for bELISA were 20.28% and 29.27%, indicating that a sample with PI % ≥ 29.27% is considered PEDV-seropositive a sample in which 20.28% < PI % < 29.27% is considered suspicious, and a sample in which PI % ≤ 20.28% is considered PEDV-seronegative.

### Specificity, sensitivity and repeatability of the blocking ELISA

**Fig. 6:**
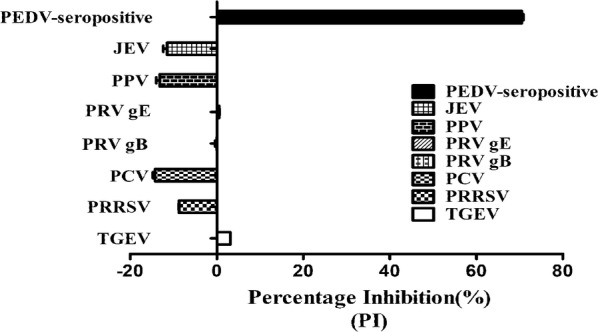
Specificity analysis of the blocking ELISA. A 96-well plate was coated with the PEDV N protein (100 ng/well) and then blocked. Dilute sera positive for 7 different pathogens (JEV, PPV, PRV-gE, PRV-gB, PCV2, PRRSV, TGEV and PEDV) were added to the wells. BiNb2 was then added to the wells, and HRP-conjugated streptavidin was added to the wells. Values are the mean PI (%) of three well replicated

Serum positive for 7 different pathogens, that are often clinically observed was used to evaluate the specificity of the blocking ELISA. PEDV-seropositive serum blocked the binding of BiNb2 to the antigen, but serum seropositive for other pathogens did not (Fig. 6). In addition, among wells coated with five different viral proteins, those coated with the PEDV N protein bound to PEDV positive serum and effectively blocked the binding of BiNb2. Although other viral proteins exhibited higher blocking rates, this was because neither PEDV serum or BiNb2 reacted with those proteins (Additional file 1: Fig. S6). This result suggests that the established bELISA exhibits high specificity.

**Fig. 7:**
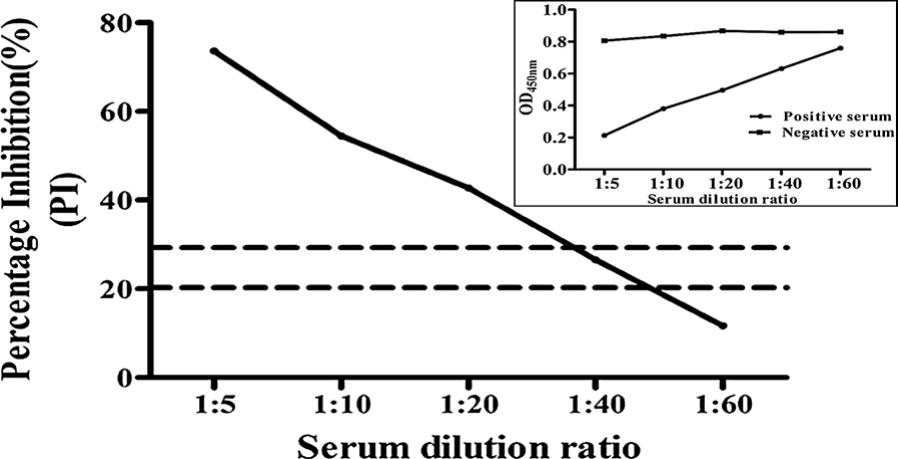
Sensitivity analysis of the blocking ELISA. A 96-well plate was coated with the PEDV N protein (100 ng/well) and then blocked. Serially diluted positive and negative sera were added to the wells and incubated at 37 °C for 2 h. Then, biNb2 was added to the wells, and HRP-conjugated streptavidin was added to the wells. Values are the mean PI (%) of three well replicated. The small figure in the upper right corner shows the OD_450nm_ value of the positive and negative sera. The dotted lines represent the upper and lower cutoff values

For further analyses of the sensitivity of the bELISA, the established bELISA was used to analyze the same PEDV-seropositive sera at different concentrations. As shown in Fig. 7, when the serum dilution factor reached 1:40, the results were considered suspicious. These results reveal that blocking ELISA can detect PEDV-seropositive serum at a highest dilution factor of 1:40, indicating the high level of bELISA sensitivity.

**Fig. 8:**
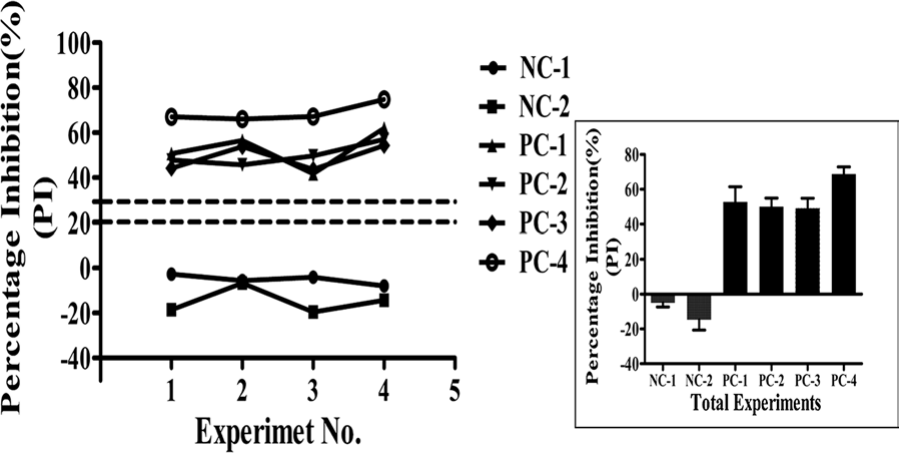
Repeatability analysis of the blocking ELISA. Intra assay repeatability was assessed using four positive and two negative sera tested on different occasions and by different operators with the newly developed bELISA. Values are the mean PI (%) of three replicate wells. The small figure shows the total average and standard deviation (SD) of four positive and negative sera across the different experiments. The dotted lines represent the upper and lower cutoff values

To further determine the repeatability of the blocking ELISA, intra-assay variability was determined by testing four replicates each of four positive serum and two negative serum sample. The bELISA displayed good repeatability, as determined by four positive and two negative serums tested on different occasions and by different operators (Fig. 8).

### Validation of the blocking ELISA

To assess whether the developed bELISA can be used in clinical samples, one hundred fifty field serum samples were detected by the developed blocking ELISA, and the results were compared to those of a commercial ELISA kit. Nineteen samples were judged to be PEDV-seropositive according to the developed blocking ELISA, and 123 samples were PEDV-seronegative, and 8 samples were considered suspicious. However, 18 samples were determined to be PEDV-seropositive with a commercial ELISA kit, and 132 samples were PEDV-seronegative. The coincidence rate between the two methods was 94% (Table 2). In addition, statistical analysis showed that the results of bELISA were highly consistent with those of the commercial ELISA kit (Kappa = 0.969); the results of the developed blocking ELISA were highly correlated with those of a commercial ELISA Kit, and the blocking ELISA was more sensitive.

**Table 2:** Comparison of the blocking ELISA and a commercialization ELISA kit

Methods and determination indexes	Blocking ELISA				Coincidence rate % (CR %)	Kappa
	Positive serum numbers	Negative serum numbers	Suspicious numbers	Total		
Commercialization ELISA						
Positive serum numbers	18	0	0	18	100	0.969
Negative serum numbers	1	123	8	132	93.18	
Total	19	123	8	150	94	

Based on the good sensitivity and specificity observed with the developed bELISA, the use of the bELISA is more advantageous than commercial kits on the market. Furthermore, the PEDV nanobody can be used with this sensitive and specific method as a high-throughput, promising diagnostic test for the detection of PEDV antiserum in farmed pigs. Compared with the monoclonal antibodies and polyclonal antibodies used in the current ELISA, the preparation of nanobodies is simpler and more inexpensive. Furthermore, the bELISA saves time and is at least an hour faster than compared to ELISA kits on the market. Therefore, bELISA is likely to be widely used in the future.

## Discussion

In the past decade, frequent outbreaks of PED caused by highly virulent PEDV strains have occurred in many swine raising countries, including those in Asia and Europe, which has caused substantial financial losses [34]. Therefore, rapid, accurate and cost–effective diagnostic methods are necessary to conduct epidemiological studies and perform immunization efficacy studies. The PEDV N protein is the best candidate antigen for early diagnosis, since it is highly conserved and pigs produce high levels of antibodies against the N protein in the early stages of PEDV infection.

Serological studies are valuable tools to investigate the prevalence and diffusion of PEDV. To date, many different types of ELISA methods have been developed, but those methods are all based on the use of expensive conventional antibodies, including polyclonal and monoclonal antibodies, are characterized by a complex production process, exhibit low yield and are costly [35, 36]. Therefore, we used nanobodies, which are the variable domains of heavy chain-only antibodies that were first discovered in camelids and sharks [37]; nanobodies can be expressed in large quantities by using prokaryotic expression systems. In the present study, we describe for the first time the successful development and validation of an Nb-based PEDV N protein blocking ELISA for the detection of antibodies in pig serum.

Here, a specific nanobody against the PEDV N protein Nb2, which specifically binds the N protein with high binding activity, was generated from a dromedary immune VHH library using a phage display technique. Notably, the specificity and sensitivity of ELISA were also determined by antibody probe. Currently, the two most commonly used antibody-probes in ELISA are horseradish peroxidase (HPR) and biotin [22, 26, 38]. There is a strong affinity between biotin and streptavidin (SA); one SA molecule can specifically bind four molecules of biotin [36, 39]. In addition, the biotin-SA complex can resist harsh environmental elements, such as proteolytic enzymes, organic solvents, and extreme temperature and pH [22]. Because of its favorable properties, the biotin-SA system is used extensively in diagnostic and therapeutic functions. Therefore, we biotinylated Nb2, a purified nanobody, fused to Avi-tag, using biotin ligase in vitro. Then, we established a new bioNb-based blocking ELISA for the detection of antibodies against PEDV.

The newly developed blocking ELISA was evaluated in terms of its specificity, sensitivity and cross-reactivity and exhibited excellent specificity and sensitivity and no cross-reactivity with serum positive for TGEV, PPV, PRRSV, PRV, JEV, PCV2 and CSFV. An intra-assay comparison also revealed the good repeatability of this method.

In addition, the newly developed blocking ELISA was compared with a commercial Porcine Epidemic Diarrhea Virus Antibody Test Kit. A total of 150 serum samples were collected from herds on several pig farms in the Shannxi and Shandong provinces from 2016 to 2018. The coincidence rate of the two methods was 94% (Table 2), indicating high correlation between the developed blocking ELISA and traditional ELISA.

## Conclusion

In this study, we successfully constructed a rich and diverse immune phage display library from which three strains of specific nanobodies of against the PEDV N protein were isolated after three consecutive rounds of bio-panning using phage display technology. Among those nanobodies, Nb2 showed good specificity and binding activity. Furthermore, a novel, rapid, specific, and low-cost blocking ELISA was developed based on biotinylated Nb2 that can be applied to assess clinical serum samples. This is the first reported case of a nanobody against PEDV and its practical application; therefore, the bELISA may become a promising diagnostic kit for the detection of PEDV antiserum in pig farms.

## Supplementary information

**Additional file 1: Table S1.** Primer pairs in the study. **Table S2.** The sensorgrams data of SPR (binding affinities of PEDV N protein to Nb2). **Fig. S1.** Titer of anti-N antibody in the immune camel serum detected by ELISA. **Fig. S2.** Construction and identification of phage library. **Fig. S3.** Specificity and binding activity of three nanobodies against PEDV N by ELISA. **Fig. S4.** Affinity analysis of PEDV N protein binding to Nbs with SPR. **Fig. S5.** Screening of bELISA conditions, including optimization of the concentration of PEDV N protein and the corresponding BiNb2 by a checkboard titration, sera dilution, serum incubation time, BiNb2 incubation time and incubation time of streptavidin-HRP on the performance of bELISA. **Fig. S6.** Specificity analysis of the bELISA by different antigens.

## Data Availability

All data generated or analyzed during this study are included in the article and additional file.
