# Supplementary material for: A novel biotinylated nanobody-based blocking ELISA for the rapid and sensitive clinical detection of porcine epidemic diarrhea virus
Source: J Nanobiotechnology. 2019 Sep 16;17:96. doi: 10.1186/s12951-019-0531-x (PMC6745792; doi:10.1186/s12951-019-0531-x)
Supplement: Supplementary file 1 — Additional file 1: Table S1. Primer pairs in the study. Table S2. The sensorgrams data of SPR (binding affinities of PEDV N protein to Nb2). Fig. S1. Titer of anti-N antibody in the immune camel serum detected by ELISA. Fig. S2. Construction and identification of phage library. Fig. S3. Specificity and binding activity of three nanobodies against PEDV N by ELISA. Fig. S4. Affinity analysis of PEDV N protein binding to Nbs with SPR. Fig. S5. Screening of bELISA conditions, including optimization of the concentration of PEDV N protein and the corresponding BiNb2 by a checkboard titration, sera dilution, serum incubation time, BiNb2 incubation time and incubation time of streptavidin-HRP on the performance of bELISA. Fig. S6. Specificity analysis of the bELISA by different antigens. [file 12951_2019_531_MOESM1_ESM.docx]

**A Novel Biotinylated Nanobody-based Blocking ELISA for the Rapid and Sensitive Clinical Detection of Porcine Epidemic Diarrhea Virus**

Zhiqian Ma,**^†^** Tianyu Wang,**^†^** Zhiwei Li, Xuyang Guo, Yangsheng Tian, Yang Li, Shuqi Xiao*

College of Veterinary Medicine, Northwest A&F University, Yangling, Shaanxi, China

*Corresponding Author : Shuqi Xiao, Tel: +86-029-87091117; Fax: +86-029-87091032

E-mail: [xiaoshuqi@nwsuaf.edu.cn](http://webmail.nwsuaf.edu.cn/web2/main.html?session_key=tkuTSaga&user=xiaoshuqi_nwsuaf_edu_cn###)

**Materials and methods**

**Serum samples**

A total of 100 standard PEDV-negative serum samples were collected from uninfected PEDV and unvaccinated pigs, and 10 standard PEDV-positive serum samples were collected from PEDV infected fattening pigs after 3 weeks. In addition, these standard serum samples were also confirmed via detection with a Porcine Epidemic Diarrhea Virus Antibody Test Kit (Biovet, Canada) and RT-PCR according to the manual instructions. Then they were used to develop the bELISA, and determine the cut-off value and specificity of the developed bELISA. To evaluate the cross reaction of the bELISA, the positive antibody samples of porcine transmissible gastroenteritis virus (TGEV), porcine parvovirus (PPV) porcine reproductive and respiratory syndrome virus (PRRSV), pseudorabies virus (PRV), Japanese encephalitis virus (JEV), porcine circovirus type 2 (PCV2), and Classical swine fever virus (CSFV) were used. Meanwhile, a total of 150 serum samples, with unknown PEDV exposure status, were collected from 5 farms from Shannxi and Shandong provinces in 2016-2018 to evaluate the validation of the bELISA.

**Camel immunization**

A 4-year-old male Bactrian camel was immunized with a mixture of highly purity 5mg of the N-His recombinant protein (1mg/mL) and an equal volume of Freund’s complete adjuvant (Sigma, USA) for the first time. To generate a highly effective immune response in camel, four additional immunizations were performed every 2 weeks with Freund’s incomplete adjuvant. A small blood sample was collected four days after the fourth and fifth immunization to evaluate the anti-N response by indirect ELISA. According to the titer result, peripheral blood lymphocytes (PBLs) were isolated from the fresh blood that was collected seven days after the fifth immunization via Ficoll-Paque PLUS (Greiner Bio-One, Germany), then the PBLs was stored at -80°C until used for RNA extraction.

**Table S1 Primer pairs in the study.**

| **Names** | **Sequences (5’-3’)** | **Reference** |
| --- | --- | --- |
| CALL001 | GTCCTGGCTGCTCTTCTACAAGG | [1] |
| CALL002 | GGTACGTGCTGTTGAACTGTTCC |  |
| VHH-FOR | GATGTGCAGCTGCAGGAGTCTGGRGGAGG | [1] |
| VHH-REV | CTAGTGCGGCCGCTGAGGAGACGGTGACCT GGGT |  |
| pET21b-VHH-F | CGGGATCCGCAGGTCCAACTGCAGGAG | [2] |
| pET21b-VHH-R | CCCAAGCTT*TTCGTGCCATTCGATTTTCTGAGCTTCGAAATATCGTTCAGACC*TGAGGAGACGGTGACCTGGGTCC |  |

1. Vincke C, Gutierrez C, Wernery U, Devoogdt N, Hassanzadeh-Ghassabeh G, Muyldermans S. Generation of single domain antibody fragments derived from camelids and generation of manifold constructs. Methods Mol Biol. 2012;907:145-76.
2. The italics sequence represents the signal peptide.

**Table S2 The sensorgrams data of SPR (binding affinities of PEDV N protein to Nb2).**

| Ligand | ka (M^−1^s^−1^) | kd (s^−1^) | ka/kd (M) |
| --- | --- | --- | --- |
| Nb1 | 1.97×10^5^ | 2.06×10^−3^ | 0.96×10^8^ |
| Nb2 | 9.21×10^5^ | 1.73×10^−3^ | 5.32×10^8^ |
| Nb3 | 3.05×10^5^ | 1.61×10^−3^ | 1.89×10^8^ |

Note: ka, kd: association and dissociation rate constants; ka/kd: the equilibrium association constant (KA).

**

**

Fig.S1 Titer of anti-N antibody in the immune camel serum detected by ELISA.





Fig.S2 Construction and identification of phage library. (A) The first round of PCR amplification products around 700bp; (B) the VHH PCR products about 400bp fragment were generated using the first purified product as templates; (C) 96 clones were randomly selected to evaluate the corrected insertion rate by bacterial fluid PCR. The size of products was about 400bp.





Fig.S3 Specificity and binding activity of three nanobodies against PEDV N by ELISA. (A) TGEV N protein and PEDV N protein were coated onto microtiter and the nanobodies were add to incubated them to tested the specificity of three nanobodies by ELISA. (B) The titer of three nanobodies and unrelated nanobody Nb12 were tested to determine of three nanobodies binding acrivity.


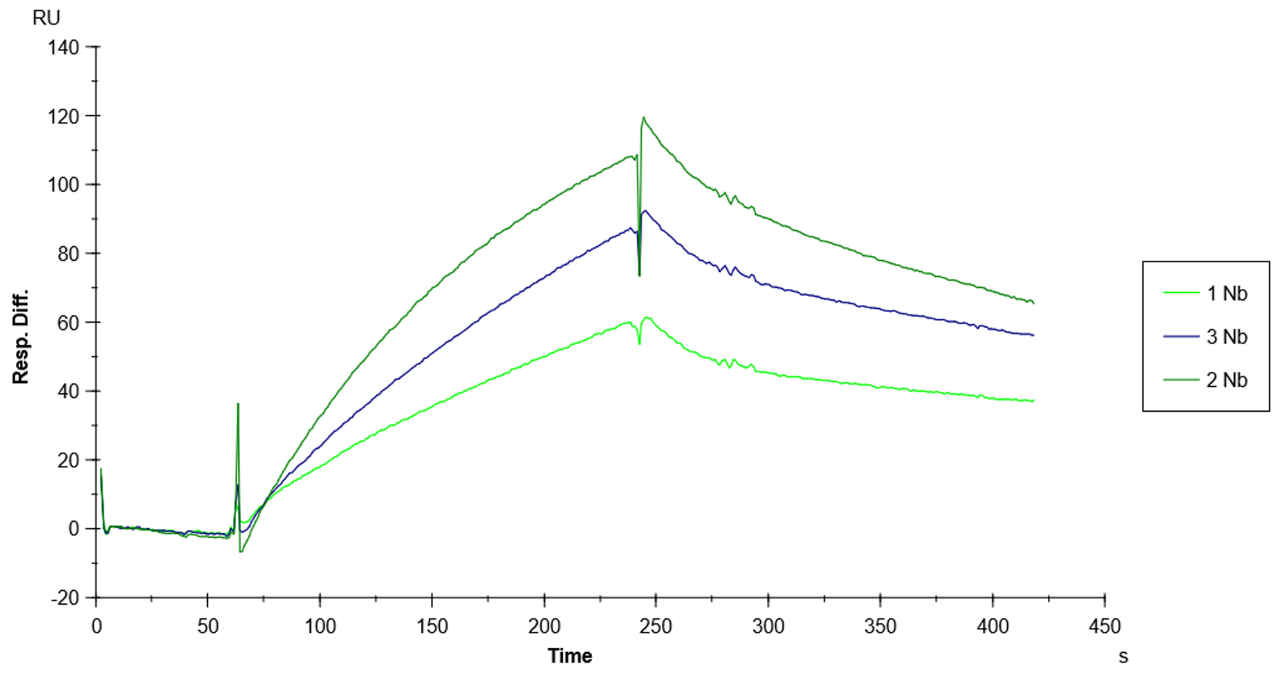


Fig.S4 Affinity analysis of PEDV N protein binding to Nbs with SPR.





Fig.S5 Screening of bELISA conditions. (A) optimization of the concentration of PEDV N protein and the corresponding BiNb2 by the checkboard titration, (B) sera dilution, (C) serum incubation time, (D) BiNb2 incubation time and (E) incubation time of streptavidin-HRP on the performance of bELISA.





Fig.S6 Specificity analysis of the bELISA. A 96-well plate was coated with the PEDV N, PEDV S, TGEV N, PRRSV N, PCV Cap and PRV gE proteins (100 ng/well) overnight at 4°C, then, the blocking ELISA were performed according to the blocking ELISA steps. Values are the mean of PI (%) of three well replicated.
